# Supplementary material for: Cytomegalovirus drug resistance mutations in transplant recipients with suspected resistance
Source: Virol J. 2023 Jul 18;20:153. doi: 10.1186/s12985-023-02127-7 (PMC10355059; doi:10.1186/s12985-023-02127-7)
Supplement: Supplementary file 1 — Supplementary Material 1 [file 12985_2023_2127_MOESM1_ESM.docx]

| SOT/HSCT | ***UL54*** | ARM_54_ | UL56 | ***UL97*** | ARM_97_ | Viral load | Antiviral | Gender | Age (y.o.) | Region |
| --- | --- | --- | --- | --- | --- | --- | --- | --- | --- | --- |
| HSCT | S |  | S | S |  | 3,36X10^3^ | GCV | FE | 56 | Galicia |
| SOT-C | ***R*** | ***F412C*** | S | ***R*** | ***C603W*** | 9,83X10^3^ | GCV and FOS | MA | 69 | Canary islands |
| HSCT | S |  | S | S |  | 6,85X10^55^ | GCV | MA | 52 | Madrid |
| HSCT | S |  | S | S |  | 8,13X10^44^ | FOS | FE | 43 | Madrid |
| SOT-K | S |  | S | S |  | 2,19X10^44^ | GCV | MA | 77 | Aragon |
| HSCT | S |  | S | S |  | 1,85X10^44^ | VGCV | MA | 51 | Madrid |
| HSCT | S |  | S | S |  | 6,91X10^3^ | VGCV | MA | 46 | Madrid |
| HSCT | S |  | S | S |  | 5,12X10^3^ | VGCV | MA | 48 | Madrid |
| HSCT | S |  | S | S |  | 3,87X10^3^ | VGCV | MA | 44 | Madrid |
| HSCT | ND |  | S | S |  | 2,85X10^3^ | VGCV | MA | 65 | Madrid |
| HSCT | ND |  | S | S |  | 8,36X10^2^ | VGCV | FE | 42 | Madrid |
| HSCT | ND |  | S | S |  | 2,93X10^3^ | VGCV | MA | 40 | Madrid |
| HSCT | ND |  | S | S |  | 4,00X10^3^ | VGCV | FE | 54 | Madrid |
| HSCT | S |  | S | S |  | 3,59X10^3^ | VGCV | FE | 45 | Madrid |
| SOT-K | ***R*** | ***L501I/ T503I/ L516R/ A834P*** | S | S |  | 3,60X10^3^ | GCV and FOS | FE | 36 | Madrid |
| HSCT | S |  | S | S |  | 5,98X10^44^ | VGCV | FE | 27 | Madrid |
| SOT-K | S |  | S | S |  | 4,30X10^3^ | GCV | MA | 70 | Galicia |
| HSCT | S |  | S | S |  | 5,07X10^44^ | VGCV | MA | 42 | Canary Islands |
| SOT-C | S |  | S | ***R*** | ***L397R/ T409M/ H411L/M460I*** | 1,00X10^55^ | GCV | MA | 62 | Galicia |
| SOT-K | S |  | S | S |  | 2,91X10^44^ |  | MA | 54 | Madrid |
| HSCT | S |  | S | S |  | 1,31X10^55^ | FOS | FE | 66 | Vasc Country |
| SOT-H | ND |  | S | S |  | 3,68X10^44^ | GCV | MA | 62 | Canary Islands |
| SOT-K | S |  | S | ***R*** | ***A594V*** | 7,29X10^44^ | GCV | MA | 59 | Galicia |
| SOT-L | S |  | S | ***R*** | ***C603W*** | 1,53X10^3^ | GCV | MA | 56 | Madrid |
| HSCT | S |  | S | S |  | 2,71X10^44^ | GCV | FE | 43 | Galicia |
| SOT-K | S |  | S | ***R*** | ***L595S/N510S*** | 1,38X10^44^ | GCV | MA | 58 | Canary Islands |
| SOT-K | ND |  | S | S |  | 3,08X10^44^ | GCV | MA | 63 | Madrid |
| HSCT | S |  | S | S |  | 3,16X10^44^ | GCV | MA | 54 | Madrid |
| SOT-K | S |  | S | S |  | 3,54X10^5^ | GCV and VGCV | MA | 67 |  |
| SOT-K | S |  | S | ***R*** | ***L595S*** | 5,92X10^44^ | GCV | MA | 58 | Madrid |
| SOT-K | S |  | S | S |  | 1,50X10^44^ | GCV | FE | 0.25 | Madrid |
| HSCT | S | S585SS | S | S |  | 4,31X10^44^ | GCV and FOS | MA | 42 | Galicia |
| HSCT | R | ***A987G*** | S | ***S*** |  | 8,74X10^3^ | VGCV, CDV | FE | 65 | Madrid |
| SOT-K | S |  | S | S |  | 1,24X10^3^ | GCV | MA | 71 | Andalucia |
| SOT-K | S |  | S | ***R*** | ***L595W*** | 4,12X10^44^ | GCV | MA | 25 | Andalucia |
| SOT-K | S |  | S | S |  | 4,17X10^4^ | GCV | MA | 69 | Andalucia |
| SOT-K | S |  | S | ***R*** | ***C607Y*** | 6,83X10^3^ | GCV | FE | 66 | Extremadura |
| SOT-H | S |  | S | S |  | 3,18X10^44^ | GCV | FE | 68 | Canary Islands |
| SOT-H | S |  | S | ***R*** | ***H520Q*** | 3,75X10^55^ | GCV | FE | 73 | Andalucia |
| SOT-L | ***R*** | ***T503I*** | S | ***R*** | ***C603W*** | 3,75X10^55^ | GCV and FOS | FE | 15 | Madrid |
| SOT-L | S |  | S | ***R*** | ***L397I*** | 2,65X10^3^ | VGCV | FE | 31 | Madrid |
| SOT-K | S |  | S | S |  | 1,80X10^3^ | GCV | MA | 24 | Andalucia |
| HSCT | S |  | S | S |  | 2,85X10^3^ | GCV | MA | 66 | Canary Islands |
| HSCT | S |  | S | S |  | 2,75X10^3^ | GCV and FOS | FE | 13 | Madrid |
| HSCT | S |  | S | S |  | 1,19X10^55^ | GCV | MA | 0.5 | Madrid |
| SOT-H | S |  | S | S |  | 5,08X10^44^ | GCV | MA | 71 | Balearic Islands |
| HSCT | S |  | S | S |  | 7,83X10^3^ | GCV and FOS | FE | 50 | Navarre |
| SOT-L | S |  | S | ***R*** | ***L595S*** | 2,84X10^3^ | GCV | FE | 0.5 | Andalucia |
| SOT-H | S |  | S | S |  | 9,86X10^3^ | GCV | MA | 54 | Andalucia |
| SOT-L | ***R*** | ***P522S*** | S | ***R*** | ***M460I/L595S*** | 6,57X10^3^ | GCV and FOS | FE | 79 | Extremadura |
| SOT-H | ND |  | S | S |  | 2,50X10^3^ | GCV | MA | 58 | Galicia |
| SOT-H | S |  | S | ***R*** | ***A594V*** | 7,85X10^3^ | VGCV and FOS | MA | 24 | Andalucia |
| SOT-C | S |  | S | ***R*** | ***L595S*** | 7,50X10^3^ | GCV | FE | 41 | Andalucia |
| HSCT | S |  | S | ***R*** | ***A594E*** | 3,85X10^3^ | VGCV | MA | 65 | Canary Islands |
| SOT-K | S |  | S | S |  | 6,24X10^3^ | VGCV | FE | 38 | Canary Islands |
| HSCT | S |  | S | S |  | 3,24X10^3^ | GCV | MA | 41 | Madrid |
| SOT-H | S |  | S | S |  | 4,4x105 | GCV | MA | 60 | Madrid |
| SOT-H | ND |  | S | S |  | 3,9x104 | GCV | MA | 59 | Madrid |
| HSCT | ND |  | S | S |  | 4,88X10^3^ | GCV | MA | 72 | Galicia |
| SOT-K | S |  | S | ***R*** | ***A594V*** | 1,45X10^44^ | VGCV | HE | 54 | Galicia |
| SOT-K | S |  | S | S |  | 9,10X10^3^ | GCV | MA | 70 | Galicia |
| HSCT | S |  | S | S |  | 6,3x104 | LET | FE | 65 | Madrid |
| SOT-H | S |  | S | S |  | 7,7x104 | VGCV | MA | 54 | Galicia |
| SOT-K | S |  | S | S |  | 5,6x104 | GCV | MA | 60 | Castille-Leon |
| SOT-k | ND |  | S | S |  | 5,7x105 | GCV | MA | 68 | Andalucia |
| SOT-L | ***R*** | ***T503I*** | S | ***R*** | ***C603W*** | 3,24X103 | VGCV and FOS | FE | 53 | Navarre |
| SOT-C | S |  | S | ND |  | 1.85 x 104 | GCV | MA | 64 | Madrid |
| SOT-H | S |  | S | ND |  | 23.9 x 103 | GCV | MA | 60 | Madrid |
| SOT-K | S |  | S | ND |  | 8.00 X10^2^ | GCV | FE | 62 | Madrid |
| SOT-L | ***R*** | ***A987G*** | S | S |  | 1,21X104 | GCV, CDV | MA | 54 | Galicia |
| HSCT | S |  | S | ND |  | 7,42X10^3^ | VGCV | MA | 59 | Madrid |
| SOT-K | S |  | S | ND |  | 1,66X10^3^ | VGCV | MA | 55 | Canary islands |
| SOT-C | S |  | S | ND |  | 2,67X10^3^ | GCV | FE | 72 | Galicia |
| SOT-C | S |  | S | ND |  | 7,70X10^3^ | GCV | MA | 52 | Galicia |
| HSCT | S |  | S | ND |  | 2,51X10^3^ | FOS | FE | 62 | Castille-Leon |
| HSCT | S |  | S | ND |  | *4,85X10*^3^ | VGCV | MA | 64 | Castille-Leon |
| SOT-K | ND |  | S | ND |  | 1,09X10^2^ | GCV | MA | 60 | Castille-Leon |
| SOT-K | ND |  | S | ND |  | 1,21X10^2^ | GCV | MA | 43 | Andalucia |
| SOT-K | ND |  | S | ND |  | 5,87X10^3^ | GCV | FE | 46 | Valencia |
| SOT-K | ND |  | S | ND |  | 2,08X10^2^ | GCV | MA | 21 | Madrid |
| SOT-K | ND |  | S | ND |  | 5,99X10^3^ | GCV | MA | 34 | Madrid |
| SOT-K | ND |  | S | ND |  | 5.65X102 | GCV | MA | 59 | Madrid |
| HSCT | ND |  | S | ND |  | 1,35X10^2^ | GCV | MA | 62 | Castille-Leon |
| SOT-K | ND |  | S | ND |  | 1,31X10^2^ | GCV | FE | 31 | Valencia |
| SOT-K | ND |  | S | ND |  | 1,12X10^2^ | GCV | FE | 76 | Castille-Leon |
| SOT-C | ND |  | S | ND |  | 4,30X10^44^ | GCV | MA | 46 | Galicia |
| SOT-K | ND |  | S | ND |  | 1,15X10^2^ | GCV | MA | 68 | Madrid |
| SOT-K | ND |  | S | ND |  | 6,93X10^3^ | GCV | MA | 33 | Madrid |
| SOT-K | ND |  | S | ND |  | 3,14X10^44^ | GCV | MA | 76 | Galicia |
| SOT-K | ND |  | S | ND |  | 1,18X10^3^ | GCV | FE | 62 | Andalucia |
| SOT-K | ND |  | S | ND |  | 1,14X10^55^ | GCV | MA | 63 | Galicia |
| SOT-K | ND |  | S | ND |  | 1,23X10^3^ | FOS | FE | 45 | Madrid |
| SOT-k | ND |  | S | ND |  | 1,30X10^3^ | GCV | MA | 61 | Madrid |
| HSCT | ND |  | S | ND |  | 5,93X10^3^ | GCV | MA | 57 | Canary islands |
| SOT-K | ND |  | S | ND |  | 3,35X10^3^ | GCV | MA | 67 | Castille-La Mancha |
| SOT-K | ND |  | S | ND |  | 1,30X10^2^ | GCV | MA | 65 | Andalucia |
